# Risk of Stroke, Myocardial Infarction, and Death Among Patients With Retinal Artery Occlusion and the Effect of Antithrombotic Treatment

**DOI:** 10.1167/tvst.10.11.2

**Published:** 2021-09-01

**Authors:** Nanna Vestergaard, Christian Torp-Pedersen, Henrik Vorum, Kristian Aasbjerg

**Affiliations:** 1 Department of Ophthalmology, Aalborg University Hospital, Aalborg, Denmark; 2 Department of Cardiology, North Zealand Hospital, Hillerød, Denmark; 3 Department of Clinical Medicine, Aalborg University, Aalborg, Denmark; 4 Himmerland Eye Clinic, Aalborg, Denmark

**Keywords:** retinal artery occlusion, stroke, antithrombotic treatment, myocardial infarction, death

## Abstract

**Purpose:**

To evaluate the risk of future stroke, myocardial infarction (MI), and death of patients with retinal artery occlusion (RAO) and the effect of various antithrombotic treatments as secondary prevention.

**Methods:**

This cohort study was based on nationwide health registries and included the entire Danish population from 2000 to 2018. All patients with RAO were identified and their adjusted risks of stroke, MI, or death in time periods since RAO were compared with those of the Danish population. Furthermore, antithrombotic treatment of patients with RAO was determined by prescription claims, and the association with the risk of stroke, MI, or death was assessed using multivariate Poisson regression models and expressed as rate ratios (RR) with 95% confidence intervals (95% CIs).

**Results:**

After inclusion, 6628 individuals experienced a first-time RAO, of whom 391 had a stroke, 66 had a MI, and 402 died within the first year after RAO. RAO was associated with an increased risk of stroke, MI, or death which persisted for more than 1 year for all three outcomes but was highest on days 3 to 14 after RAO for stroke, with an adjusted RR of 50.71 (95% CI, 41.55–61.87), and on days 14 to 90 after RAO for MI and death, with adjusted RRs of 1.98 (95% CI, 1.25–3.15) and 1.64 (95% CI, 1.28–189), respectively. Overall, antithrombotic treatment was not associated with any protective effect the first year.

**Conclusions:**

Patients with RAO had an increased risk of stroke, MI, or death. No protective effect of antithrombotic treatment was shown.

**Translational Relevance:**

These findings are relevant to the management of patients with RAO.

## Introduction

Acute retinal artery occlusion (RAO) often causes severe and irreversible visual loss in affected patients and may be regarded as the ocular analog to cerebral stroke. The majority of RAO is caused by embolism, the most common sources being the carotid artery and the heart.^1^ Despite the severity of the disease, an effective treatment remains elusive. For the management of acute RAO, various treatment strategies have been suggested, of which thrombolytics has received special attention. However, a retrospective study from 2010 did not find any difference among these treatments and observations.^2^ The lack of effect of fibrinolysis in RAO is still debated and under further investigation.^3^^–^^5^

For patients with RAO, the prevalence of diabetes, hypertension, and smoking is higher compared with controls,^1^ and similarly their risk of future cardiovascular events has been a subject of interest. Indeed, previous studies have suggested that patients with RAO are at increased risk of stroke, but evidence regarding their risk of myocardial infarction (MI) and death is more sparse.^6^^–^^12^ Due to this apparent high risk of future vascular diseases, a large emphasis on secondary prevention of systemic ischemic events would seem prudent. According to the American Stroke Association, RAO is defined as an ischemic stroke; hence, the recommendations on the management and treatment with antiplatelet therapy or anticoagulation in stroke should apply to patients with RAO, as well.^13^ The use of antiplatelet therapy is effective in reducing the risk of recurrent stroke for patients with stroke^14^; however, only very limited research has been published on RAO and the use of antiplatelet therapy in the prevention of future vascular events.^15^

Hence, this study seeks to enlighten the risk of future stroke, MI, or mortality for patients with RAO and the preventive effect of antithrombotic treatment.

## Method

### Study Design

This study is a retrospective cohort study based on Danish nationwide register data and consists of two main analysis: (1) the risk of stroke, MI, or death for patients with RAO compared with the Danish population as the baseline, and (2) assessment of the effect of antithrombotic treatment by stratifying patients with RAO based on their use of antithrombotic medication and comparing their risk of future events with untreated patients with RAO.

### Registries

At birth or immigration, every citizen in Denmark is assigned a unique, permanent civil registration number, allowing linkage of individuals among nationwide registries. Of these registries, the Danish National Patient Register holds information on all hospital admissions and outpatient visits in Denmark, and hospitals are required by law to report these data to receive public funding. The data include dates and all relevant diagnoses at discharge classified according to the International Classification of Diseases, either the 8th or 10th revision before or after 1994, respectively (ICD-8 or ICD-10). The National Prescription Register holds information on all dispensed drug prescriptions from pharmacies in Denmark, and these are coded according to the Anatomical Therapeutic Chemical (ATC) classification system. The information includes tablet strength and number of tablets and packs. In Denmark, pharmacies are required by law to report all dispensations uniquely linked to the individual in order to manage drug reimbursement. Information on sex and date of birth is obtained from the National Population Register, and deaths are registered in the National Cause of Death Register.

### Ethical Considerations

In Denmark, registry-based studies do not require ethical approval. This study was approved and registered by the data-responsible institute (Region Hovedstaden; approval number P-2019-348) in accordance with the General Data Protection Regulation.

### Study Population

The study cohort was comprised of all Danish patients with a hospital diagnosis of RAO (either branch or central RAO; ICD-10 H34.1 or H34.2), and the entire Danish population was included as a baseline. Individuals were assigned as patients with RAO 3 days after their first RAO diagnosis. This was done for the following three reasons: (1) to allow time for the first prescription of antithrombotic medication to be claimed, as there is often a delay from disease to claimed prescription; (2) to allow for a washout of these tablets for 3 days to correctly identify those in treatment, as patients may very well be given a few tablets of antithrombotics by the hospital but only reimbursed drugs appear in the Prescription Register; and (3) to ensure that a concurrent event happening at the time of the RAO was not included, as this study sought to investigate the risk of future events.

As the risk of vascular events is correlated to time since the RAO, the analysis was stratified in time intervals: 3 to 14 days, 14 to 90 days, 90 to 365 days, or more than 365 days after RAO. Inclusion started at year 2000 and continued until December 2018. Individuals were included at the age of 30 years and were followed until the occurrence of any of the following, whichever came first: death, outcome, or December 31, 2018.

### Comorbidities

The following comorbidities were included as potential confounders: diabetes mellitus, hypertension, heart failure, chronic kidney disease, cancer, ischemic heart disease, stroke, and atrial fibrillation. See Supplementary Table S1 and S2 for ICD and ATC codes. Diabetes mellitus was defined as either (1) hospital diagnosis or (2) one claimed prescription of glucose-lowering drug. Likewise, hypertension was defined as either (1) hospital diagnosis or (2) claimed prescriptions from at least two of six drug classes in a period of 90 days.

### Outcome

Separate analyses were performed to calculate the risk of each outcome. These were defined as a hospital diagnoses of stroke or MI or as the occurrence of all-cause death.

### Antithrombotic Treatment

To analyze the effect of antithrombotic treatment, a second analysis was carried out in which patients with RAO were stratified according to their use of antithrombotic treatment. Hence, patients with RAO were stratified into the following subgroups: (1) no treatment; (2) aspirin (ATC B01AC06); (3) clopidogrel (ATC B01AC04); (4) anticoagulant treatment including warfarin (ATC B01AA03), phenprocoumon (ATC B01AA04), dabigatran (ATC B01AE07), edoxaban (ATC B01AF03), rivaroxaban (ATC B01AF01), or apixaban (ATC B01AF02); or (5) other antiplatelet treatment including ticagrelor (ATC B01AC24) and prasugrel (ATC B01AC22).

Treatment status was determined in a time-dependent manner based on dispensed drug prescriptions. This was done to account for treatment initiated before RAO and variations in time from RAO to start of treatment; hence, patients were considered as being on treatment only when covered by a prescription claim. Coverage was calculated by an algorithm on the basis of tablet strength and number of tablets in each prescription held against a standard daily dosage for that specific medication, as done previously.^16^ After multiple consecutive claims, an individual daily dosage was determined, with respect to predefined maximum, minimum, and most typical dosages. In case of remaining tablets, it was presumed they were consumed in the period immediately after the ending of the last prescription coverage.

Moreover, patients with RAO were defined as treatment naive if they had not claimed any prescription of antithrombotic treatment 2 years prior to their RAO. The proportion of these patients starting treatment after RAO was investigated.

### Statistical Analysis

The risk ratios (RRs) of stroke, MI, and death were analyzed in a time-dependent way by using multivariate Poisson regression. To do this, all individuals were split into multiple records according to three time scales (calendar time, age, and time since RAO). In the first analysis in which the Danish population was included as the baseline, all individuals contributed with disease-free time until the occurrence of RAO. In the second analysis, on the effect of antithrombotic treatment, patients with RAO contributed with treatment time when covered by a prescription claim and untreated time when they were not—the latter being defined as the reference.

Separate analyses were carried out for each outcome. The analyses were adjusted for sex and age, diabetes mellitus, hypertension, heart failure, calendar time, chronic kidney disease, cancer, ischemic heart disease, stroke, and atrial fibrillation (AFLI) modeled as time-dependent exposure variables. Moreover, the second analysis concerning the effect of treatment was adjusted for whether the patients with RAO had received any antithrombotic treatment 2 years prior to their RAO (treatment naive or not). The analyses were stratified in time intervals since RAO. Incidence RRs were reported with 95% confidence intervals (CIs). *P* < 0.05 was considered significant.

Due to the study design, descriptive statistics were carried out only on patients with RAO. They were grouped by their use of antithrombotic treatment at any time during the first 3 to 90 days after RAO, and their characteristics were defined when starting their treatment. Patients receiving different antithrombotic treatments during this period were included in multiple groups. Hence, the treatment groups are not exclusive. The baseline characteristics of the total group of patients having a RAO after inclusion were defined at the time of the RAO.

For graphical presentation, the cumulative risks accounting for competing risk (death) were estimated for stroke and myocardial infarction, whereas all-cause mortality was expressed by Kaplan–Meier estimates. To construct a comparison group, patients with RAO were matched (exposure density matching) in a 1:4 ratio with individuals from the Danish population based on age (rounded to 10) and sex.

All data management and statistical analyses were performed using R (R Foundation for Statistical Computing, Vienna, Austria).^17^

### Other Analyses

A subgroup analysis on the effect of antithrombotic treatment was performed with censoring on a number of conditions. Hence, patients were excluded if they had temporal arteritis or AFLI or had undergone a carotid endarterectomy, as the treatment of these patients differs from that for the main group of patients with RAO.^18^ Moreover, patients with ischemic heart disease or previous stroke are at increased risk of vascular events and should be considered for antithrombotic treatment, regardless of the occurrence of RAO. The same applies to patients already receiving antithrombotic treatment. Therefore, as RAO most likely will not influence the clinical decision on whether to start antithrombotic treatment, these patients were censored in the subgroup analysis.

For the Poisson analyses, the assumption of constant risk in intervals was tested by demonstrating nearly identical results with splits at 1-year intervals for calendar year and age. Moreover, sensitivity analyses were performed on the second main analysis in which (1) the inclusion started in 2007 instead, and (2) only temporal arteritis and carotid endarterectomy were excluded.

## Results

In total, 5,158,279 Danish citizens were included from 2000 to 2018, of whom 6628 individuals had a first-time RAO after inclusion. Of these patients with RAO, 3340 (50.4%) were treatment naive (i.e., had received no antithrombotic treatment in 2 years prior to the RAO). Almost half of the treatment-naive patients with RAO (*n* = 1489; 44.6%) claimed a prescription of antithrombotic treatment in the first 90 days after their RAO and before the occurrence of any of the outcomes. However, this changed over time, as in year 2000 only 27.2% started treatment, increasing gradually to 60.6% in 2018. Moreover, for the large majority, the treatment was initiated with some delay from the RAO. By the time of inclusion of patients with RAO at 3 days after the RAO, 23.3% (*n* = 778) of the treatment-naive patients had claimed their first prescription. The basic characteristics of the included patients with RAO are presented in the Table, in which they are grouped by their use of antithrombotic treatment during the first 3 to 90 days after RAO. The large majority of the treated patients received aspirin (3144 patients) followed by clopidogrel (1443 patients). Compared with untreated patients with RAO, those in any of the treatment groups generally were older and more likely to be male and have any of the comorbidities.

**Table.** Basic Characteristics of RAO Patients Grouped by Antithrombotic Treatment Received Any Time the First 3 to 90 Days After RAO

	Treatment					
Characteristic	Aspirin (*n* = 3144)	Clopidogrel (*n* = 1443)	Anticoagulant (*n* = 723)	Other Antiplatelet (*n* = 34)	Untreated (*n* = 1820)	Total (*n* = 6628)
Age (yr), mean (SD)	71.1 (11.7)	70.6 (11)	73 (11.4)	69.2 (11.2)	66.3 (14.2)	69.8 (12.5)
Female sex, *n* (%)	1476 (47.0)	622 (43.1)	274 (37.9)	10 (29.4)	853 (47.5)	3006 (45.5)
Diabetes, *n* (%)	547 (17.4)	277 (19.2)	157 (21.7)	12 (35.3)	218 (12.0)	1086 (16.4)
Hypertension, *n* (%)	951 (30.2)	516 (35.8)	360 (49.8)	16 (47.1)	280 (15.4)	1859 (28.0)
Heart failure, *n* (%)	279 (8.9)	107 (7.4)	191 (26.4)	6 (17.6)	68 (3.7)	562 (8.5)
Chronic kidney disease, *n* (%)	201 (6.4)	92 (6.4)	89 (12.3)	0 (0.0)	76 (4.2)	401 (6.1)
Cancer, *n* (%)	472 (15.0)	254 (17.6)	140 (19.4)	6 (17.6)	217 (11.9)	984 (14.8)
Atrial fibrillation, *n* (%)	233 (7.4)	65 (4.5)	471 (65.1)	4 (11.8)	63 (3.5)	699 (10.5)
Ischemic heart disease, *n* (%)	835 (26.6)	403 (27.9)	331 (45.8)	29 (85.3)	185 (10.2)	1547 (23.3)
Any antithrombotic treatment 2 years prior to the RAO, *n* (%)	2053 (65.3)	819 (56.8)	631 (87.3)	29 (85.3)	185 (10.2)	3288 (49.6)

The treatment groups are not exclusive. The untreated group includes patients with RAO who did not receive any antithrombotic treatment at any time during the first 3 to 90 days after RAO. The total group includes all patients having RAO after inclusion.

### Risk of Future Stroke, MI, or Death

**Figure 1.**
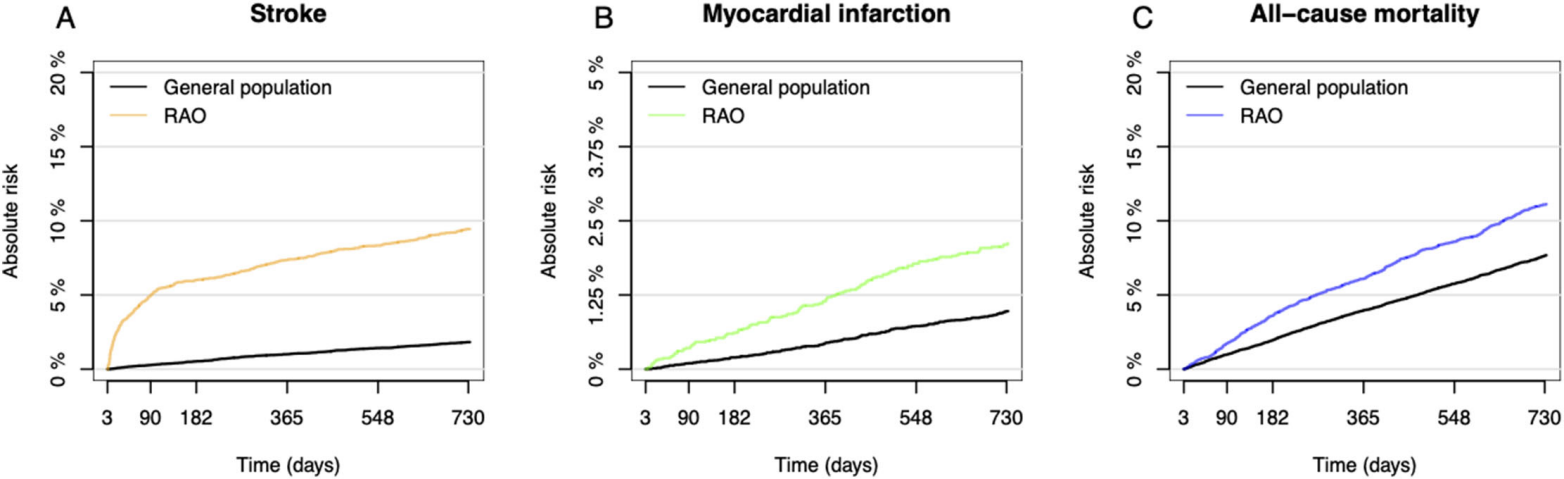
(A–C) Cumulative risk of stroke and myocardial infarction accounting for competing risk and Kaplan–Meier curves for all-cause mortality. For patients with RAO, time corresponds to days after the RAO, starting at day 3.

**Figure 2.**
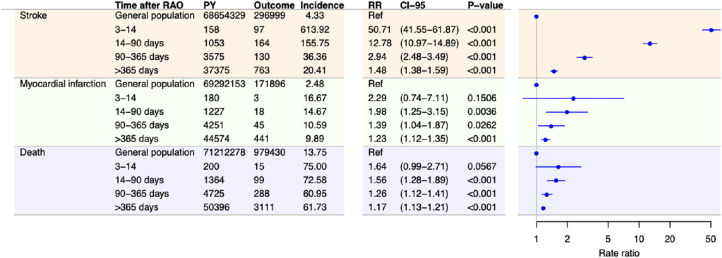
Incidence and adjusted RRs for stroke, MI, and death among patients with RAO compared with the general Danish population stratified in time periods after RAO. RRs are adjusted for sex, age, calendar time, diabetes mellitus, hypertension, heart failure, chronic kidney disease, cancer, atrial fibrillation, ischemic heart disease (only the analysis on stroke and death), and stroke (only the analysis on death and MI). Incidences are per 1000 person years. PY, person years; CI-95, 95% confidence interval.

As depicted in Figures 1 and 2, patients with RAO had higher risks of stroke, MI, or all-cause death compared with the general population. The greatest increase in risk was seen for stroke, for which the increased risk persisted in all time intervals after RAO but was highest during the first 3 to 14 days, with an adjusted RR of 50.71 (95% CI, 41.55–61.87; *P* < 0.001). For MI and death, the risks were significantly higher for patients with RAO after 14 days.

### Effect of Antithrombotic Treatment

**Figure 3.**
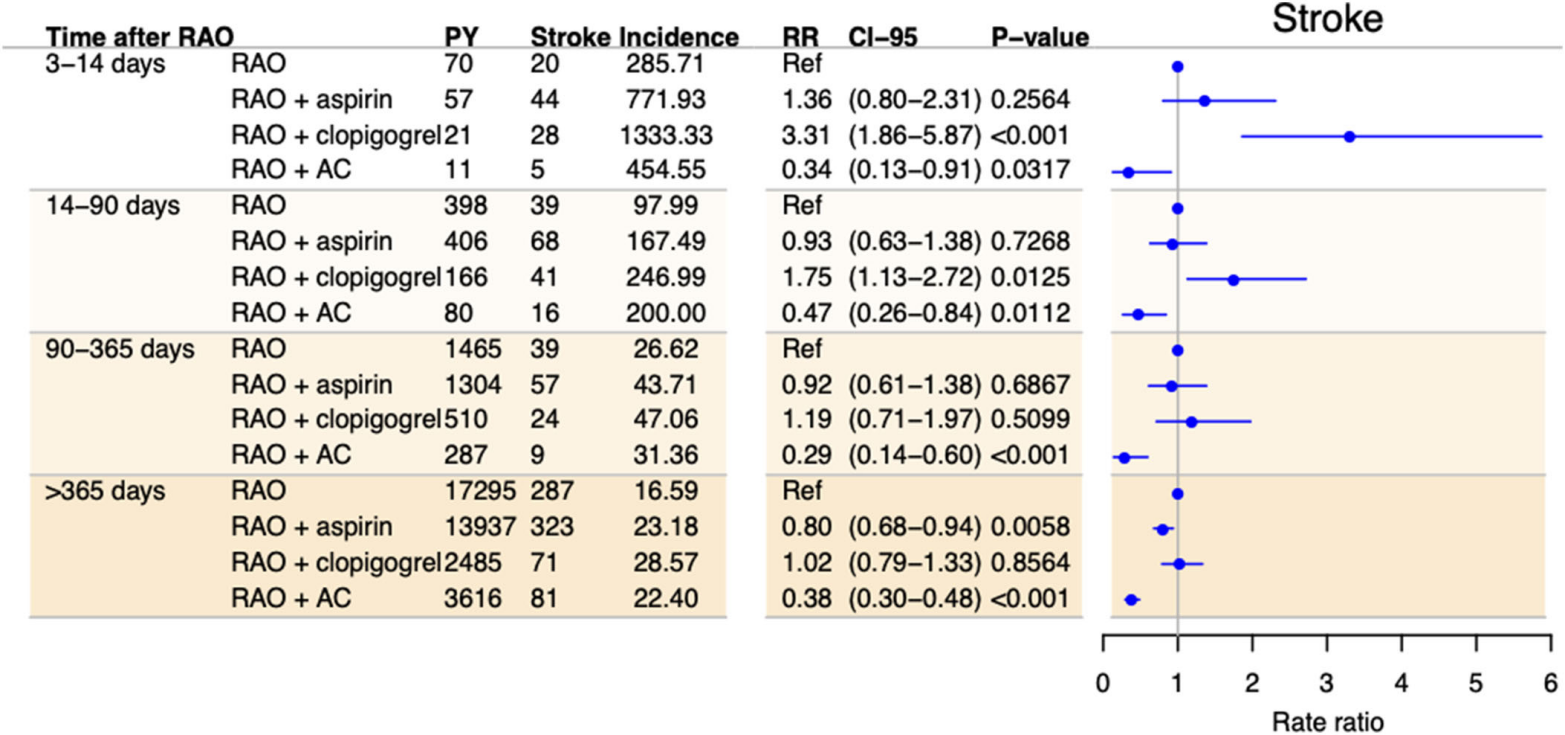
Incidence and adjusted RRs of stroke among patients with RAO in time periods after RAO stratified by treatment with antithrombotic medication, with no treatment as the reference. Rate ratios are adjusted for sex, age, calendar time, diabetes mellitus, hypertension, heart failure, chronic kidney disease, cancer, atrial fibrillation, ischemic heart disease, and previous antithrombotic treatment. Incidences are per 1000 person years. AC, anticoagulant treatment.

The results from the analysis on the effect of antithrombotic treatment are presented in Figures 3^4^ to 5. Overall, treatment with aspirin did not change the risk of any of the outcomes the first year, but after 1 year treatment with aspirin was associated with a small decrease in the risk of both stroke (adjusted RR, 0.80; 95% CI, 0.68–0.94; *P* = 0.0058) and death (adjusted RR, 0.86; 95% CI, 0.79–0.93; *P* < 0.001).

**Figure 4.**
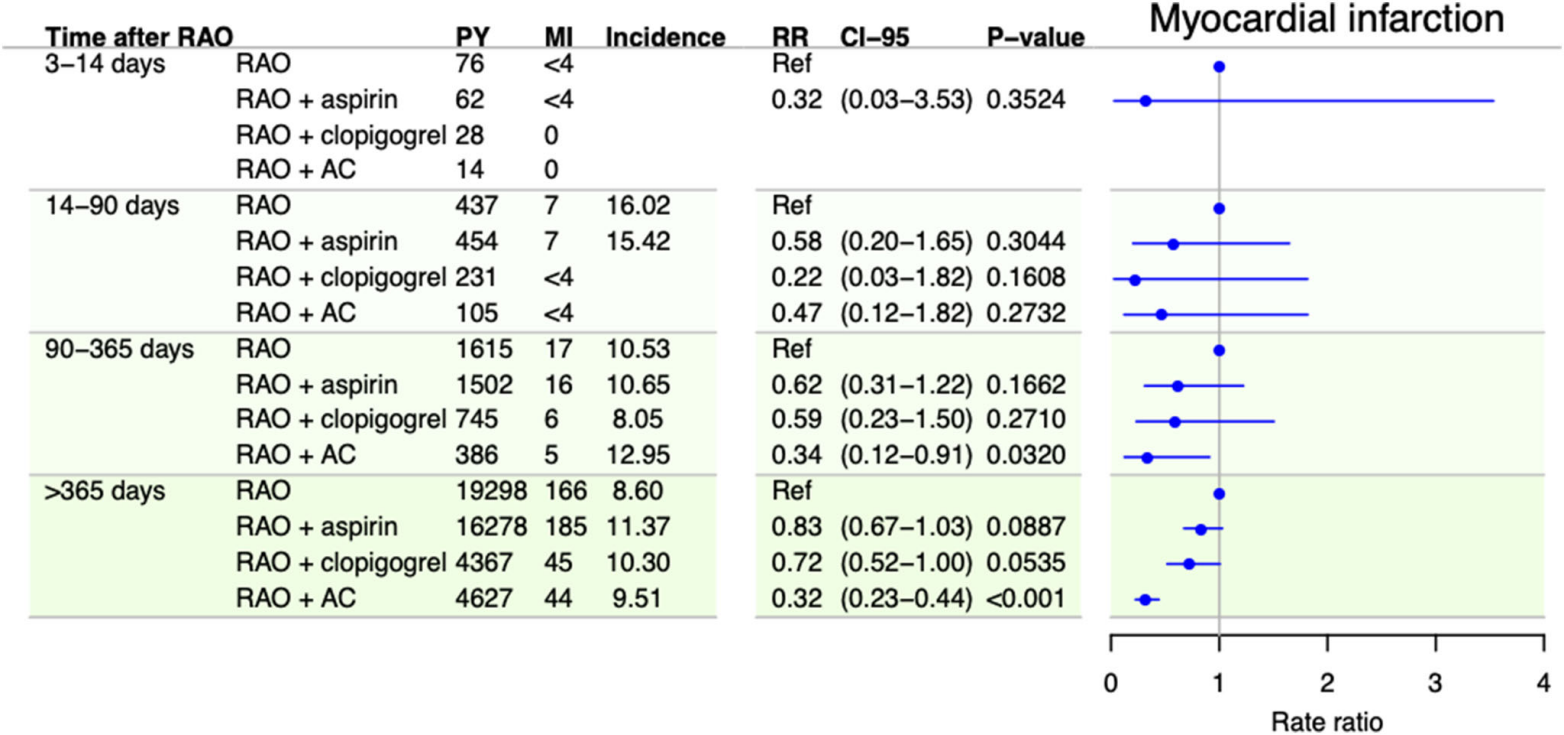
Incidence and adjusted RRs of myocardial infarction among patients with RAO in time periods after RAO stratified by treatment with antithrombotic medication, with no treatment as the reference. RRs are adjusted for sex, age, calendar time, diabetes mellitus, hypertension, heart failure, chronic kidney disease, cancer, atrial fibrillation, stroke, and previous antithrombotic treatment. Incidences are per 1000 person years.

**Figure 5.**
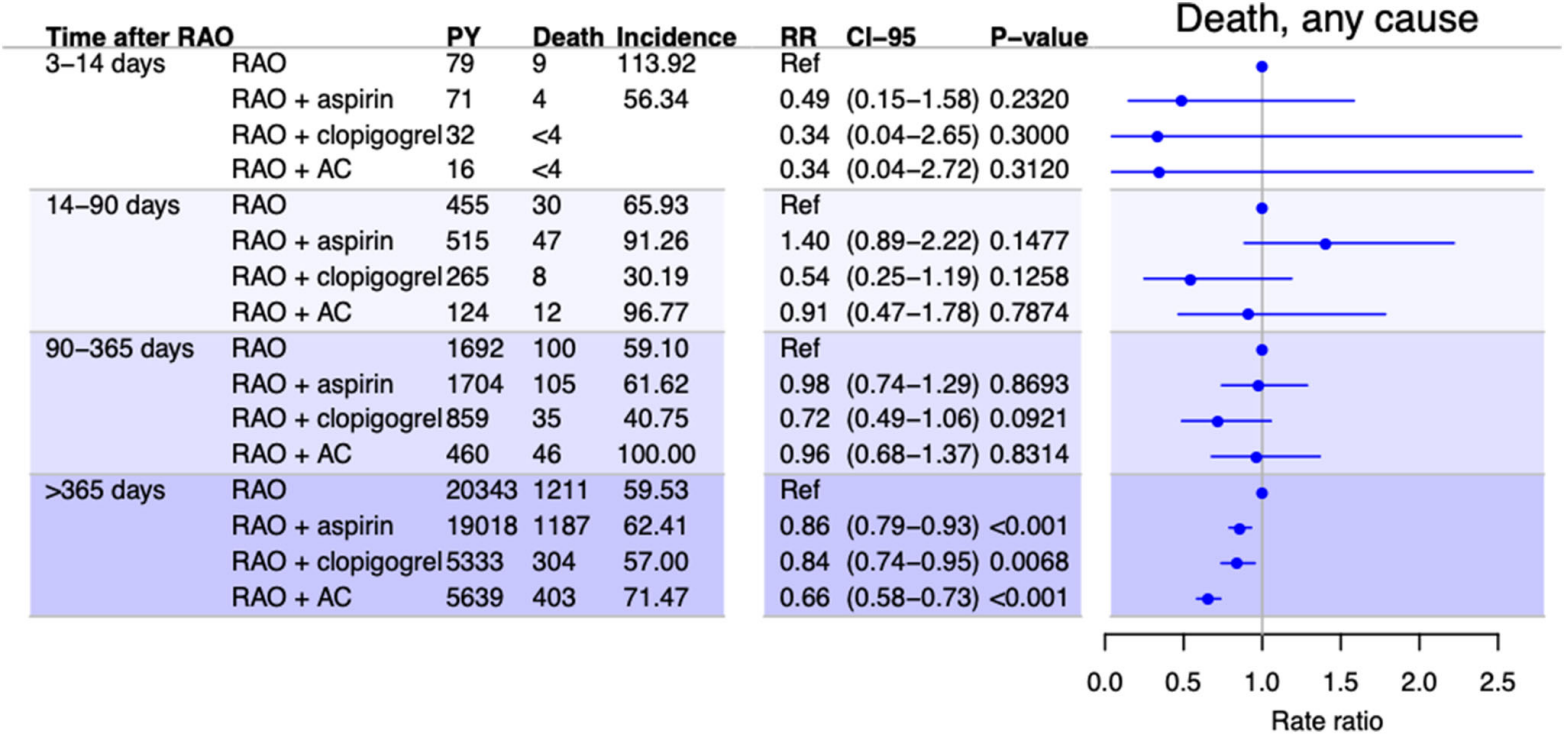
Incidence and adjusted RRs of all-cause death among patients with RAO in time periods after RAO stratified by treatment with antithrombotic medication, with no treatment as the reference. RRs are adjusted for sex, age, calendar time, diabetes mellitus, hypertension, heart failure, chronic kidney disease, cancer, atrial fibrillation, ischemic heart disease, stroke, and previous antithrombotic treatment. Incidences are per 1000 person years.

Clopidogrel was associated with a decreased risk of death after 1 year, with an adjusted RR of 0.84 (95% CI, 0.74–0.95; *P =* 0.0068). No protective effect was observed for any of the other outcomes in either of the analyses. In contrast, clopidogrel was associated with an increased risk of stroke for days 3 to 14 and days 14 to 90. Anticoagulant treatment was associated with a decreased risk of stroke in all time periods and of MI and death after 90 and 365 days, respectively. The sample size of patients treated with antiplatelets other than aspirin or clopidogrel was too small to perform meaningful statistical analyses. Sensitivity analyses were performed to test the robustness of the analyses. The results are presented in Supplementary Figures S1 and S2.

#### Subgroup Analyses

The results from the subgroup analyses are presented in Supplementary Figure S3. These analyses included only patients with RAO who had not received previous antithrombotic treatment and did not have any of the following conditions: temporal arteritis, AFLI, carotid endarterectomy, previous stroke, or ischemic heart disease. Generally, these results were in line with the main analyses with some exceptions. After 1 year, aspirin was associated with a decreased risk of only death and not of stroke. Moreover, anticoagulant treatment was not associated with a protective effect in any of the outcomes.

## Discussion

This large nationwide cohort study included more than 6600 patients with RAO and found RAO to be associated with an increased risk of stroke, MI, or death, with the risk for stroke being most prominent. The increased risk was markedly higher right after RAO but persisted after 1 year.

Generally, the results showed no consistent protective effect of any of the antithrombotic treatments, but after 1 year aspirin and clopidogrel treatment might be associated with a small decrease in risk. To some extent, anticoagulant treatment was associated with a decreased risk of the outcomes, but, because the subgroup analyses did not confirm this result, the protective effect is most likely only true for a small group of patients with RAO with known cardiovascular diseases such as atrial fibrillation and hence cannot be generalized.

About half of the patients did not receive antithrombotic treatment prior to their RAO. Only about half of these treatment-naive patients with RAO started antithrombotic treatment in the first 90 days after their RAO, with an increasing tendency in the more recent years.

The increased and time-correlated risk of stroke found in the present study is in line with the large majority of existing studies.^4^^,^^19^^,^^20^ A recent review and meta-analysis based on magnetic resonance imaging scans of mainly neurological asymptomatic patients with RAO found that as many as 30% of patients with RAO and 25% of patients with RAO had acute cerebral ischemia in the first 7 days after RAO.^21^ Similar results were found in another recent paper.^22^ However, as RAO is a rather rare disease, previous studies are comprised mainly of smaller cohort studies and only a few larger, register-based studies in Asian populations. Thus, a 2012 Taiwan register-based cohort study by Chang et al.^23^ found the incidence of stroke in patients with RAO to be almost 10 times higher compared with matched controls in the first month after the event. However, these findings were not adjusted for confounders such as diabetes. Similarly, a Korean population-based study from 2015 by Park et al.^11^ found the incidence RR of stroke to be 21.50 for patients with RAO the first 30 days after the event compared with control periods in the same patients before and/or after the time around the event. Another Korean study found that the 10-year incidence of stroke was increased for patients with RAO, but unfortunately the analysis was not stratified in time intervals after.^12^ As these studies were all carried out in Asian populations, there is a limitation in their generalizability to other ethnic groups. Another population-based study included the US Medicare population and used patients with hip fractures as the control group. They found that the incidence RR of stroke was increased only the first 2 weeks after RAO. Unfortunately, their results were not adjusted for any comorbidities.^24^ Our study adds to the existing knowledge as it includes a European population. Furthermore, stratification into three time intervals showed that the risk was excessive even after the first 7 days and persisted for more than a year. Hence, although the prompt initiation of preventive treatment is important, treatment is still relevant even after a substantial delay.

In contrast to most other studies, a small study by Leisser and Findl^25^ including 30 patients only found the risk of stroke to be increased in patients with previous stroke, transient ischemic attack, and/or amaurosis fugax. Likewise, a recent study by Laczynski et al.^26^ did not find an increased risk of stroke among patients with RAO. Based on findings from their own registries they question the findings from large register-based studies in general. The authors highlighted (1) the accuracy of stroke diagnoses in registries in general, as they found only a minority of the stroke diagnoses to be correct in their own registry; and (2) the possibility that a concurrent stroke at the time of RAO diagnosis could be misinterpreted with regard to the timing of the two events. Although we do recognize the importance of validation of registries and the possible limitations of registries in general, the extrapolation of characteristics of one registry to others seems rather unjustified. In the Danish National Registry of Patients, the registration of MI and acute stroke has been validated and found to have positive predictive values of 97% and 79% to 93.5%, respectively.^27^^,^^28^ Moreover, in this study patients with RAO were only assigned as such 3 days after the RAO to ensure that any registered stroke occurred after the RAO.

Compared to stroke, the risks of MI and death among patients with RAO have received much less attention.^29^ A study on coronary computed tomography angiography in patients with RAO and controls with no cardiac symptoms found a higher prevalence of obstructive coronary artery disease among patients with RAO.^30^ Indeed, a register-based Taiwan study from 2015 found a hazard ratio of 1.72 for developing acute coronary symptoms in patients with RAO compared with controls, but it was not further analyzed in terms of time since the RAO.^31^ In this regard, the study by Park et al.^11^ did not find the relative risk of MI to be increased around the time of the RAO compared with the general risk in these same patients.^11^ In contrast, this present study found the risk of MI to be correlated to time since RAO, although the effect was markedly lower compared with stroke, as would be expected. In contrast to stroke, MI and RAO are not caused by the same embolic source. Instead, the correlation is more likely a result of common systemic diseases such as atherosclerosis.

As the risks of MI, stroke, or death are highest immediately following RAO, a protective effect of antiplatelets could provide the greatest benefit during this period. However, our study did not find any protective effect the first year after RAO. The effect of aspirin on the risk of stroke the first year after RAO was also investigated in a recent register-based cohort study from Taiwan.^15^ They found a high risk of stroke immediately after the RAO but were unable to find any preventive effect of aspirin. Their study included only 15 stroke events the first 90 days in the aspirin group (37 in the non-treatment group), whereas this study included 96 and 51 events, respectively. In contrast to our study, the Taiwan study defined treatment solely based on the prescription of aspirin on the event day, leading to less than 13% of the patients with RAO being defined as the treatment group for 1 year as opposed to our cohort, in which around half of the treatment-naive patients with RAO started treatment within 90 days. In our cohort, there was a delay from RAO to the initiation of treatment in the vast majority of patients starting treatment in the first 90 days. In fact, fewer than one-third of these treatments were initiated the first few days. By applying a time-dependent approach, a very precise definition of the treatment group was carried out.

Despite these differences, both studies have come to the same conclusion of no effect the first year, and to our knowledge there are no other similar studies.

In contrast, a meta-analysis including 195 randomized controlled trials found that treatment with aspirin or another antiplatelet agents reduced the risk of MI, stroke, or vascular deaths among high-risk patients in general.^32^ As with other similar studies, the effects for patients with RAO specifically were not evaluated. The report did state, however, that, “… the protective effects of antiplatelet therapy should be expected to apply to an even wider range of high risk patients than those categories for which the present meta-analysis provides direct evidence of benefit.”^32^ Even if the effect of antiplatelet treatment is smaller in other patients, such as patients with RAO, the hazards of antiplatelet treatment are still considered to be outweighed by their effect.^32^

Because large-artery atherosclerosis, most often in the form of stenoses or plaques in the carotid arteries, is the most frequent cause of RAO and these patients may have a higher risk of subsequent thromboembolic events compared with other patients with RAO,^7^^,^^33^ evidence concerning patients with carotid atherosclerosis should be relevant to patients with RAO. The Clinical Practice Guidelines of the European Society for Vascular Surgery from 2017 recommend antiplatelet treatment in symptomatic patients with a 50% to 99% stenosis even if not undergoing surgery.^18^ However, these recommendations are also largely based on extrapolation from general stroke populations. To our knowledge, the presumed effect of antiplatelets as secondary prevention in patients with RAO has not been investigated specifically.

The reasons for the discrepancies between these studies and our study on patients with RAO remain uncertain. One possible explanation is that the emboli causing RAO and a subsequent stroke are of a type and composition different from those causing classic strokes. This argument is raised by some authors to explain why thrombolytics does not work in the acute management of RAO, as it has been shown that the majority of retinal emboli are made of cholesterol or calcified material.^3^ This would explain why our results suggest that there might be a protective effect after 1 year, as these events due to the lack of timely correlation are less likely to result from the exact same source of emboli but rather from general atherosclerosis. However, these findings were not confirmed in the subgroup analysis with respect to stroke and MI.

If instead the emboli causing subsequent stroke resembles those produced in atrial fibrillation, then one should expect anticoagulants to be more effective than antiplatelet treatment, which was indeed the case in the main analysis in our study. As discussed, the subgroup analyses did not confirm this result, but we cannot exclude that it is affected by confounding by indication.

### Strengths and Limitations

The study design allowed the inclusion of a large number of patients with RAO despite its being a rare disease, thus making it possible to stratify on both treatment status and time since the event. Furthermore, it empowered an analysis of the less frequent events, death and MI. As the analyses were time dependent with continuous updates on the treatment status based on actual prescriptions, the treatment groups were defined very accurately.

This study also has several limitations. Primarily, we were unable to control for smoking status, body mass index, and family history; therefore, we cannot exclude the possibility of confounding by indication (i.e., more diseased patients with a higher risk of the outcome receiving antithrombotic treatment and thereby masking an effect of the antithrombotic medications). This could explain why we found an increased risk of stroke associated with the use of clopidogrel. Furthermore, it is not possible to draw any conclusion on the severity of strokes. Hence, a previous study found patients with atherothrombotic strokes treated with prestroke aspirin to have milder symptoms from their stroke and a better outcome after 3 months.^34^ Finally, the diagnosis of RAO has not been validated, and due to our study design we cannot draw any conclusions on the first 3 days after RAO. However, as it is a very specific diagnosis made by ophthalmologists only, we expect it to be valid. We do not have data on visual outcome. To draw any final conclusions on the efficacy of antithrombotic treatment, a randomized controlled trial is needed.

## Conclusions

This study of more than 6600 patients with RAO found RAO to be associated with an increased risk of stroke, MI, or death. The increased risk of vascular events after RAO further emphasizes the importance of immediate preventive treatment. However, this study did not find any protective effect of antithrombotic treatments in the first year after RAO, in which the risk of vascular events is highest.

## Supplementary Material

Supplement 1

Supplement 2

Supplement 3
