# Supplementary material for: Risk of Stroke, Myocardial Infarction, and Death Among Patients With Retinal Artery Occlusion and the Effect of Antithrombotic Treatment
Source: Transl Vis Sci Technol. 2021 Sep 1;10(11):2. doi: 10.1167/tvst.10.11.2 (PMC8419877; doi:10.1167/tvst.10.11.2)
Supplement: Supplement 1 [file tvst-10-11-2_s001.pdf]

Supplementary 1

Table 1S. Classification of diseases based on hospital diagnoses (ICD codes). ICD = International Classification of Diseases 8th or 10th revision (before or after 1994)

Disease	ICD-8	ICD-10
Diabetes	250	E10-14
Hypertension	40009, 40019, 40029, 40039, 40099, 40199, 40299, 40399, 40499	I10-15
Heart failure	42599, 42709, 42710, 42711, 42719	I142, I50, I110, J81
Chronic kidney disease	N02, N03, N04, N05, N06, N07, N08, N11, N12, N14, N18, N19, N26, N158, N159, N160, N162, N163, N164, N168, Q61, E102, E112, E132, E142, I120, M321B, Q612, Q613, Q615, Q619	24902, 25002,58200, 58201, 58202, 58208, 58209, 58300, 58301, 58302, 58308, 58309, 58499, 59009, 59320, 75310, 75311, 75319, 79299, 40399, 40499, 44609, 44629
Cancer	109-140	C00-9, C10-97
Ischemic heart disease	41009, 41099, 41109, 41199, 41209, 41299, 41309, 41399, 41409, 41499	I20-25
Myocardial infarction	41009, 41099	I21, I22
Stroke (including TCI)	43309, 43399, 43409, 43499, 43600, 43601, 43609, 43690, 43699, 43700, 43701, 43708, 43709, 43790, 43791, 43798, 43799, 43809, 43899, 43509, 43599	I63, I64, G458, G459

Table 2S. Classification of diseases based on medication (ATC codes). Diagnosis of hypertension is based on the use of at least 2 different drug classes (column 2). ATC = Anatomical Therapeutic Chemical Classification System.

Disease	Group of medication	ATC
Diabetes	Antidiabetics	A10
Hypertension	Anti adrenergic	C02A, C02B, C02C
	Diuretics	C02DA, C03A, C03B, C03D, C03E, C03X, C02DA, C07B, C07C, C07D, C08G, C09BA, C09DA, C09XA52, C02L
	Vasodilator	C02DB, C02DD, C02DG
	Beta blocker	C07A, C07B, C07C, C07D, C07F
	Calcium antagonist	C07FB, C08, C08G, C09BB, C09DB
	Renin angiotensin inhibitor	C09AA, C09BA, C09BB, C09CA, C09DA, C09DB, C09XA02, C09XA52
