# Supplementary material for: Risk of Stroke, Myocardial Infarction, and Death Among Patients With Retinal Artery Occlusion and the Effect of Antithrombotic Treatment
Source: Transl Vis Sci Technol. 2021 Sep 1;10(11):2. doi: 10.1167/tvst.10.11.2 (PMC8419877; doi:10.1167/tvst.10.11.2)
Supplement: Supplement 2 [file tvst-10-11-2_s002.pdf]

Supplementary 2

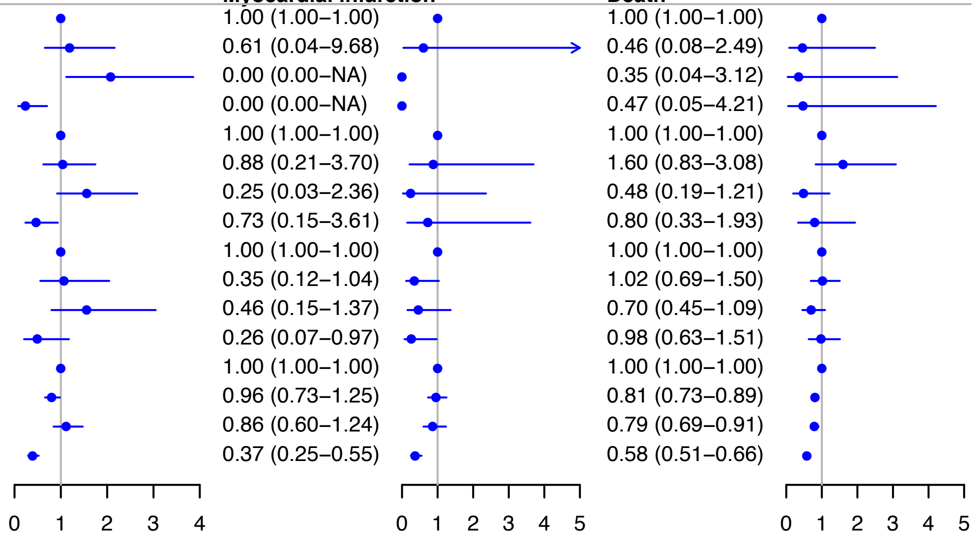

Time after RAO		Stroke	Myocardial infarction	Death
3–14 days	RAO	1.00 (1.00–1.00)	1.00 (1.00–1.00)	1.00 (1.00–1.00)
	RAO + aspirin	1.19 (0.66–2.16)	0.61 (0.04–9.68)	0.46 (0.08–2.49)
	RAO + clopigogrel	2.08 (1.12–3.86)	0.00 (0.00–NA)	0.35 (0.04–3.12)
	RAO + AC	0.23 (0.08–0.70)	0.00 (0.00–NA)	0.47 (0.05–4.21)
14–90 days	RAO	1.00 (1.00–1.00)	1.00 (1.00–1.00)	1.00 (1.00–1.00)
	RAO + aspirin	1.04 (0.62–1.75)	0.88 (0.21–3.70)	1.60 (0.83–3.08)
	RAO + clopigogrel	1.56 (0.92–2.65)	0.25 (0.03–2.36)	0.48 (0.19–1.21)
	RAO + AC	0.47 (0.23–0.94)	0.73 (0.15–3.61)	0.80 (0.33–1.93)
90–365 days	RAO	1.00 (1.00–1.00)	1.00 (1.00–1.00)	1.00 (1.00–1.00)
	RAO + aspirin	1.07 (0.56–2.05)	0.35 (0.12–1.04)	1.02 (0.69–1.50)
	RAO + clopigogrel	1.56 (0.80–3.05)	0.46 (0.15–1.37)	0.70 (0.45–1.09)
	RAO + AC	0.49 (0.21–1.17)	0.26 (0.07–0.97)	0.98 (0.63–1.51)
>365 days	RAO	1.00 (1.00–1.00)	1.00 (1.00–1.00)	1.00 (1.00–1.00)
	RAO + aspirin	0.80 (0.66–0.98)	0.96 (0.73–1.25)	0.81 (0.73–0.89)
	RAO + clopigogrel	1.11 (0.84–1.47)	0.86 (0.60–1.24)	0.79 (0.69–0.91)
	RAO + AC	0.39 (0.29–0.52)	0.37 (0.25–0.55)	0.58 (0.51–0.66)

Figure S1. Results from sensitivity analyses in which the inclusion started in year 2007 depicted as rate ratios of stroke, myocardial infarction, and death among RAO patients in time periods after RAO stratified on treatment with antithrombotic medication with no treatment as the reference. Results are adjusted for sex, age, calendar time, diabetes mellitus, hypertension, heart failure, chronic kidney disease, cancer, atrial fibrillation, previous antithrombotic treatment, ischemic heart disease (the analyses on stroke and death), and stroke (the analyses on myocardial infarction and death).

AC = anticoagulant treatment, RAO = retinal artery occlusion.

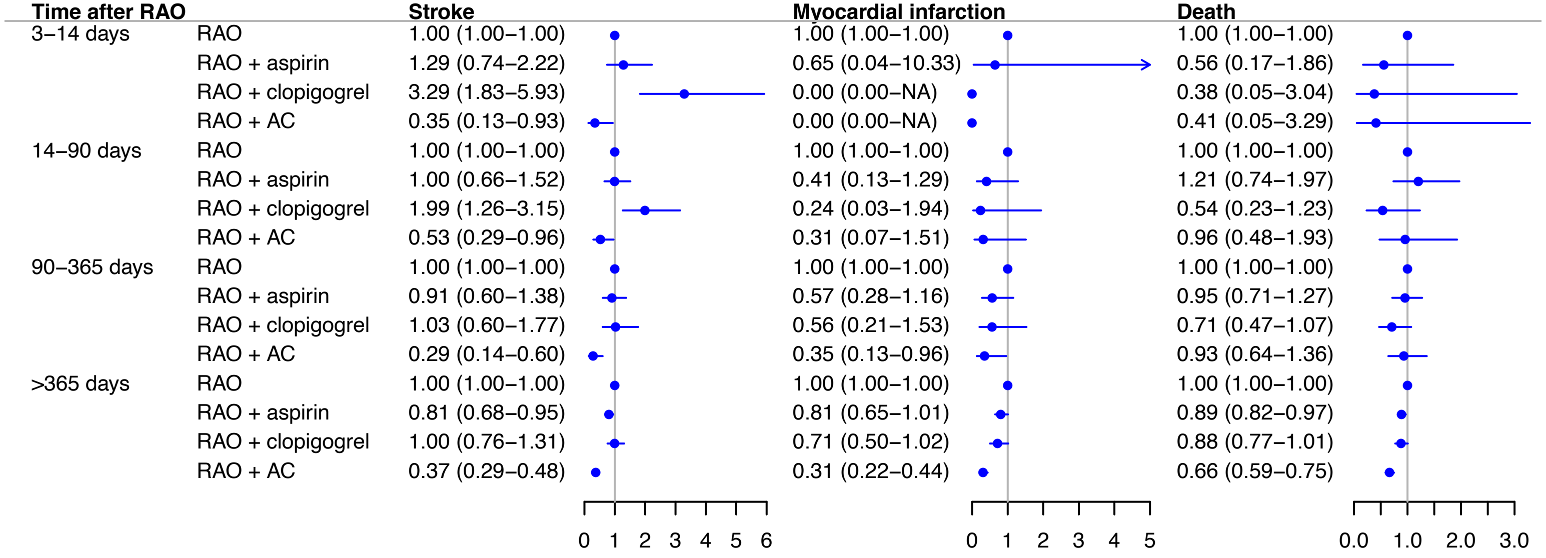

Time after RAO		Stroke	Myocardial infarction	Death
3–14 days	RAO	1.00 (1.00–1.00)	1.00 (1.00–1.00)	1.00 (1.00–1.00)
	RAO + aspirin	1.29 (0.74–2.22)	0.65 (0.04–10.33)	0.56 (0.17–1.86)
	RAO + clopigogrel	3.29 (1.83–5.93)	0.00 (0.00–NA)	0.38 (0.05–3.04)
	RAO + AC	0.35 (0.13–0.93)	0.00 (0.00–NA)	0.41 (0.05–3.29)
14–90 days	RAO	1.00 (1.00–1.00)	1.00 (1.00–1.00)	1.00 (1.00–1.00)
	RAO + aspirin	1.00 (0.66–1.52)	0.41 (0.13–1.29)	1.21 (0.74–1.97)
	RAO + clopigogrel	1.99 (1.26–3.15)	0.24 (0.03–1.94)	0.54 (0.23–1.23)
	RAO + AC	0.53 (0.29–0.96)	0.31 (0.07–1.51)	0.96 (0.48–1.93)
90–365 days	RAO	1.00 (1.00–1.00)	1.00 (1.00–1.00)	1.00 (1.00–1.00)
	RAO + aspirin	0.91 (0.60–1.38)	0.57 (0.28–1.16)	0.95 (0.71–1.27)
	RAO + clopigogrel	1.03 (0.60–1.77)	0.56 (0.21–1.53)	0.71 (0.47–1.07)
	RAO + AC	0.29 (0.14–0.60)	0.35 (0.13–0.96)	0.93 (0.64–1.36)
>365 days	RAO	1.00 (1.00–1.00)	1.00 (1.00–1.00)	1.00 (1.00–1.00)
	RAO + aspirin	0.81 (0.68–0.95)	0.81 (0.65–1.01)	0.89 (0.82–0.97)
	RAO + clopigogrel	1.00 (0.76–1.31)	0.71 (0.50–1.02)	0.88 (0.77–1.01)
	RAO + AC	0.37 (0.29–0.48)	0.31 (0.22–0.44)	0.66 (0.59–0.75)

Figure S2. Results from sensitivity analyses in which individuals were excluded if they had temporal arteritis or had performed carotid endarterectomy, depicted as rate ratios of stroke, myocardial infarction, and death among RAO patients in time periods after RAO stratified on treatment with antithrombotic medication with no treatment as the reference. Results are adjusted for sex, age, calendar time, diabetes mellitus, hypertension, heart failure, chronic kidney disease, cancer, atrial fibrillation, previous antithrombotic treatment, ischemic heart disease (the analyses on stroke and death), and stroke (the analyses on myocardial infarction and death).

AC = anticoagulant treatment, RAO = retinal artery occlusion.
