# Supplementary material for: Risk of Stroke, Myocardial Infarction, and Death Among Patients With Retinal Artery Occlusion and the Effect of Antithrombotic Treatment
Source: Transl Vis Sci Technol. 2021 Sep 1;10(11):2. doi: 10.1167/tvst.10.11.2 (PMC8419877; doi:10.1167/tvst.10.11.2)
Supplement: Supplement 3 [file tvst-10-11-2_s003.pdf]

Supplementary 3

A

Time after RAO		PY	Stroke	Incidence	RR	CI-95	P-value
3–14 days	RAO	58	17	293.10	Ref		
	RAO + aspirin	38	29	763.16	2.04	(1.12–3.71)	0.0196
	RAO + clopigogrel	14	23	1642.86	5.15	(2.75–9.63)	<0.001
	RAO + AC	2	<4				
14–90 days	RAO	328	29	88.41	Ref		
	RAO + aspirin	279	51	182.80	1.66	(1.05–2.62)	0.0295
	RAO + clopigogrel	110	25	227.27	2.34	(1.37–4.00)	0.0018
	RAO + AC	14	5	357.14	3.17	(1.23–8.19)	0.0172
90–365 days	RAO	1201	24	19.98	Ref		
	RAO + aspirin	881	31	35.19	1.35	(0.79–2.29)	0.2748
	RAO + clopigogrel	333	13	39.04	1.63	(0.83–3.21)	0.1545
	RAO + AC	56	<4		0.65	(0.09–4.80)	0.6729
>365 days	RAO	14234	193	13.56	Ref		
	RAO + aspirin	8838	169	19.12	0.97	(0.79–1.20)	0.7908
	RAO + clopigogrel	1448	34	23.48	1.27	(0.89–1.84)	0.1919
	RAO + AC	688	14	20.35	0.95	(0.55–1.64)	0.8651

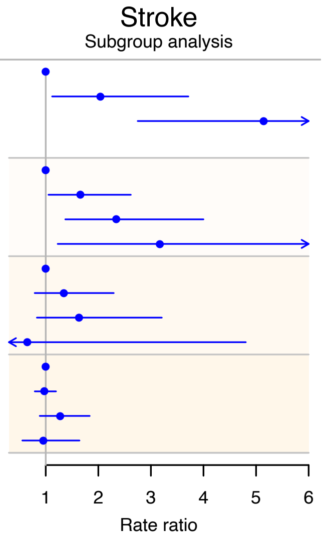

B

Time after RAO		PY	MI	Incidence	RR	CI-95	P-value
3–14 days	RAO	63	<4		Ref		
	RAO + aspirin	45	<4		0.98	(0.06–15.69)	0.9895
	RAO + clopigogrel	17	0				
	RAO + AC	3	0				
14–90 days	RAO	355	5	14.08	Ref		
	RAO + aspirin	326	5	15.34	0.83	(0.24–2.87)	0.7714
	RAO + clopigogrel	129	<4		0.50	(0.06–4.28)	0.5267
	RAO + AC	20	0				
90–365 days	RAO	1303	12	9.21	Ref		
	RAO + aspirin	1029	11	10.69	0.82	(0.36–1.86)	0.6400
	RAO + clopigogrel	399	<4		0.21	(0.03–1.59)	0.1295
	RAO + AC	79	<4		1.90	(0.54–6.74)	0.3196
>365 days	RAO	15377	107	6.96	Ref		
	RAO + aspirin	10678	102	9.55	0.89	(0.68–1.17)	0.4208
	RAO + clopigogrel	1828	20	10.94	0.91	(0.56–1.47)	0.6974
	RAO + AC	1085	11	10.14	0.64	(0.34–1.19)	0.1554

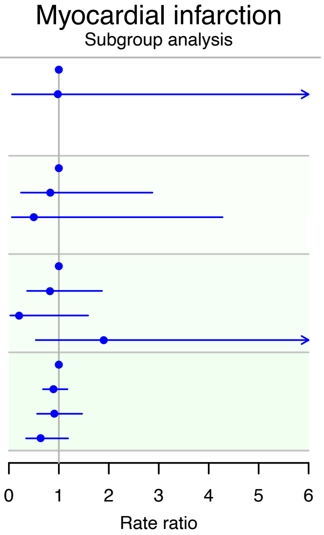

C

Time after RAO		PY	Death	Incidence	RR	CI-95	P-value
3–14 days	RAO	58	<4		Ref		
	RAO + aspirin	38	<4		0.40	(0.04–3.88)	0.4321
	RAO + clopigogrel	14	<4		1.73	(0.18–16.59)	0.6365
	RAO + AC	2	<4				
14–90 days	RAO	328	11	33.54	Ref		
	RAO + aspirin	279	14	50.18	1.28	(0.58–2.81)	0.5460
	RAO + clopigogrel	110	<4		0.32	(0.04–2.45)	0.2712
	RAO + AC	14	<4		1.97	(0.25–15.25)	0.5166
90–365 days	RAO	1201	39	32.47	Ref		
	RAO + aspirin	881	24	27.24	0.69	(0.41–1.14)	0.1492
	RAO + clopigogrel	333	5	15.02	0.48	(0.19–1.21)	0.1184
	RAO + AC	56	<4		1.37	(0.42–4.44)	0.5967
>365 days	RAO	14234	460	32.32	Ref		
	RAO + aspirin	8838	301	34.06	0.78	(0.68–0.90)	<0.001
	RAO + clopigogrel	1448	33	22.79	0.65	(0.46–0.93)	0.0186
	RAO + AC	688	29	42.15	1.08	(0.74–1.57)	0.6822

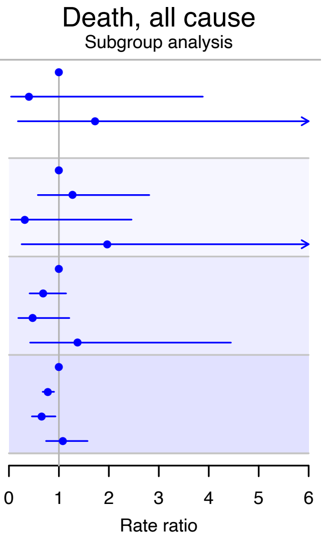

Figure 3S, A-C. Results from the subgroup analyses including only RAO patients who had not received previous antithrombotic treatment and did not have any of the following conditions: temporal arteritis, AFLI, carotid endarterectomy, previous stroke or IHD. Results presented as incidence and rate ratios of stroke, myocardial infarction, and death respectively in time periods after RAO stratified on treatment with antithrombotic medication with no treatment as the reference. Rate ratios are adjusted for sex, age, calendar time, diabetes mellitus, hypertension, heart failure, chronic kidney disease, and cancer. Incidences are per 1000 person years.

AC = anticoagulant treatment, PY = person years, MI = myocardial infarction, RR = rate ratio, CI-95 = 95% confidence interval, RAO = retinal artery occlusion.
